# Automatic pest identification system in the greenhouse based on deep learning and machine vision

**DOI:** 10.3389/fpls.2023.1255719

**Published:** 2023-09-28

**Authors:** Xiaolei Zhang, Junyi Bu, Xixiang Zhou, Xiaochan Wang

**Affiliations:** College of Engineering, Nanjing Agricultural University, Nanjing, China

**Keywords:** tiny pest detection, improved YOLOv5, pest population dynamics, pest trapping system, greenhouse

## Abstract

Monitoring and understanding pest population dynamics is essential to greenhouse management for effectively preventing infestations and crop diseases. Image-based pest recognition approaches demonstrate the potential for real-time pest monitoring. However, the pest detection models are challenged by the tiny pest scale and complex image background. Therefore, high-quality image datasets and reliable pest detection models are required. In this study, we developed a trapping system with yellow sticky paper and LED light for automatic pest image collection, and proposed an improved YOLOv5 model with copy-pasting data augmentation for pest recognition. We evaluated the system in cherry tomato and strawberry greenhouses during 40 days of continuous monitoring. Six diverse pests, including tobacco whiteflies, leaf miners, aphids, fruit flies, thrips, and houseflies, are observed in the experiment. The results indicated that the proposed improved YOLOv5 model obtained an average recognition accuracy of 96% and demonstrated superiority in identification of nearby pests over the original YOLOv5 model. Furthermore, the two greenhouses show different pest numbers and populations dynamics, where the number of pests in the cherry tomato greenhouse was approximately 1.7 times that in the strawberry greenhouse. The developed time-series pest-monitoring system could provide insights for pest control and further applied to other greenhouses.

## 1 Introduction

Monitoring pest population dynamics are essential to greenhouse management for effectively predicting the potential distribution of pests and preventing infestations and crop diseases (Deng et al., 2018; Capinha et al., 2021; Preti et al., 2021). Therefore, monitoring the time series of pest numbers in greenhouse is important to support human pest control decisions (Wen et al., n.d). However, automatic identification of pest populations in greenhouses is challenging due to the small size of pests. The current manual pest detection and counting approaches are labor intensive, time-consuming, and unreliable (Xie et al., 2018). Developing a fast and reliable automatic pest identification and counting approach may reduce the workload and improve timely pest control (Rustia et al., 2021b).

Image-based automatic pest monitoring systems can replace laborious manual identification and improve reliability, thus proving to be a powerful tool with real-time pest information to facilitate agricultural management (Sun et al., 2017; Rustia et al., 2020; Wang et al., 2020a). Deep learning has shown the potential of image processing with end-to-end feature extraction patterns for automatic pest identification and counting (Liu and Wang, 2020; Liu and Wang, 2021). To build an accurate deep learning model for automatic pest identification, high-quality and large-scale image datasets are indispensable (Li and Chao, 2021; Li et al., 2021b).

Current pest image datasets are mainly acquired from the internet, crop surface, pest-trapping containers, or sticky boards (**Table 1**). Pest datasets collected from the internet typically contain a large number of samples and various categories. A previous study reported an IP 102 dataset containing 75,000 images of 102 pest species collected from agricultural and insect-science websites; the dataset was evaluated using machine learning models including support vector machine, K Nearest Neighbors, and deep learning methods like Faster R-CNN, and FPN (Wu et al., 2019). Another website-based dataset for crop pest recognition comprises 46,567 images from 41 classes (Wang et al., 2022). However, online datasets have limited practical pest-monitoring applications because many pest categories are not representative of greenhouse crop production and show low-level harm to common crops (Wang et al., 2021).

**Table 1:** Current pest image collection approaches and pest detection models.

Image collection approach	Pest identification model	# Pest species	Application scenarios	Year	References
Internet-based	CaffeNet	9	Paddy	2018	(Alfarisy et al., 2018)
	SVM, KNN, AlexNet, GoogleNet, VGGNet, ResNet	102	/	2019	(Wu et al., 2019)
	EffcientNetB0, ResNet50, GoogleNet, ShuffleNet, MobileNetv2, DenseNet201	122	/	2022	(Nanni et al., 2022)
Crop surface	Unsupervised feature learning	40	Corn, soybean, wheat, and canola	2018	(Xie et al., 2018)
	SSD512, RetinaNet, FCOS, Faster R-CNN, FPN, Cascade R-CNN	14	Wheat, rice, corn, and rape	2021	(Wang et al., 2021)
	SNIPER, ClusDet, DMNet, DCTDet, DCTDet + YOLOv3, etc.	1	Wheat	2022	(Du et al., 2022)
Trapping containers	RetinaNet	6	Pine forests	2018	(Sun et al., 2018)
	ResNet	5	Paddy	2020	(Yao et al., 2020)
	YOLOv5	2	Paddy and vineyard	2023	(Teixeira et al., 2023)
Sticky boards	Faster RCNN, SSD, YOLOv3, and Cascade R-CNN	24	Field crops	2020	(Wang et al., 2020b)
	CNN	4	Tomato and lisianthus greenhouse	2020	(Rustia et al., 2021a)
	Faster R-CNN, TPest-RCNN	2	Green pepper greenhouse	2021	(Li et al., 2021a)
	YOLOv5	4	Witloof chicory	2023	(Kalfas et al., 2023)

“/” means data not available.

Field investigations have mainly focused on pest images collected from crop fields with specific illumination conditions, crops, or pest types. A current study developed of an AgriPest dataset including 14 common pests of wheat, rice, corn, and rape (Wang et al., 2021). The filed collected datasets usually demonstrated different pest distribution density, light reflection variability, crop surface backgrounds, and pest types (Xie et al., 2018; Du et al., 2022). However, pest detection models demonstrate high precision in the present datasets, but are not replicable in a greenhouse setting due to large variations in pest type, environmental conditions, and unclear image collection owing to the pest movement. This limits the application of powerful deep learning technology for pest control in specific domains such as greenhouses.

Pest images collected with trapping devices are less affected by the environmental conditions and plant types. Trapping containers adopt pheromones, toxic gas, or a lamp to attract pests, and collect the image from its baseplate (Li et al., 2021b). Capturing pest images by light trapping have been widely used for automatic monitoring of pests (Wang et al., 2021). However, the trapping containers designed based on the pest phototaxis typically use a concentrated light source, thus pests are attracted near the light source with overlapping, which is not applicable to long-term monitoring. A vibration plate and a moving conveyor belt could be adopted to disperse the pests and avoid overlapping (Yao et al., 2020). The trapping containers usually used for detecting large size pests, but not effective for tiny pests of high-density in the greenhouse.

A Sticky board with color tropism can collect clear pest images by attracting flying pests, which are widely used as trapping devices (Ding and Taylor, 2016; Sütő, 2021). It reduces pest overlapping and limb mutilation, and could be applied to pests of different sizes. Current studies reported automatic methods for detecting insect pests using sticky paper traps and achieved high counting accuracies (Li et al., 2021a; Rustia et al., 2021a). The trapping board-based systems, however, have low trapping efficiency at night, and thus could not accurately monitoring pest number changes over 24 hours. In addition, changes in light during the day and night cause significant variations in the images in the trapping board, which reduce the accuracy and reliability of pest detection. Therefore, it is essential to develop a trapping system with a uniform light source and a high trapping efficiency.

Considering the diversity of pests, automatic pest-recognition models are required. Deep learning, with the advantage of an end-to-end learning strategy, is the current state-of-the-art object detection approach. Recently, deep-learning-based pest detection models are developed for multiclass pest detection and classification, and achieved high performances (Liu et al., 2019; Wang et al., 2020b). However, most models are primarily designed for large-scale pest detection in field crops, which may not be directly applicable to the detection of small pests in greenhouses due to the limited number of pixels in their images; therefore, it is necessary to develop small object detection models for greenhouse pests. The YOLOv5 model was developed for high inference and at three different scales, enabling it to effectively tackle the challenges associated with small-object detection in images. (Zhan et al., 2022; Zhao et al., 2022). The mosaic data augmentation adopted in the YOLOv5 model enriches the dataset by random scaling, cutting, and arranging the original input (Zhao et al., 2022). Further, it improves the accuracy of small target identification, especially when the number of large or medium targets is higher.

To address the challenge of automatic pest detection and counting in greenhouses, a comprehensive experiment on pest image recognition was conducted in two greenhouses, a cherry tomato and a strawberry greenhouse. The objectives of this study were as follows: 1) design an automatic pest identification system with an LED lamp and insect-trapping board to trap pests and automatically capture images with a uniform background to improve the insect trapping efficiency; 2) develop an improved YOLOv5 model with copy-pasting data augmentation to effectively identify small-sized pests and improve recognition accuracy of unbalanced distributed pest images; and 3) Monitoring the pest population dynamics in two greenhouses and compare their changing patterns.

## 2 Methods

### 2.1 Experimental design

**Figure 1:**
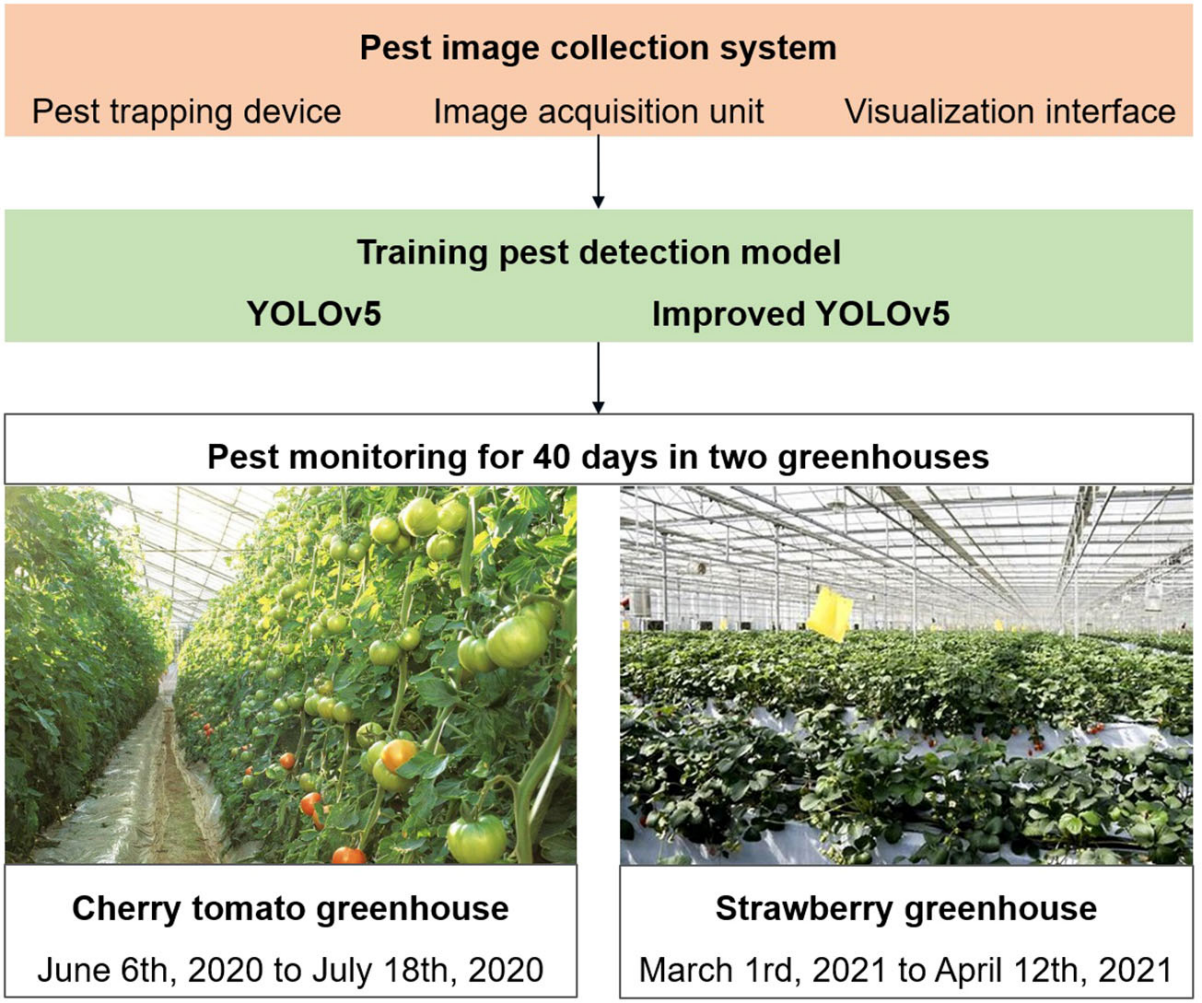
Experimental design of pest image collection, pest detection model and pest population monitoring.

We developed an automatic pest image collection system in this study. An improved YOLOv5 model was proposed for detecting small-sized pests, and its performance was compared with that of the original YOLOv5 model. Pest images were collected during the spring and summer for 43 days in two greenhouses (**Figure 1**). We examined two greenhouses in Nanjing, China. The first one is a “Hongxi” cherry tomato glass greenhouse situated in Xuanwu District, and the second one is a “Hongyan” strawberry multi-span greenhouse located in Pukou District. For the first three days, we placed 10 insect traps in the greenhouses and collected 10 images of each greenhouse to train a pest detection model. After the first three days, we used one insect trap to collect images and monitor the pest populations daily. Images were collected regularly at 16:00. Thus, we utilized 20 original images and applied data augmentation techniques to expand the training dataset to train the pest detection model and 80 images to monitor pest populations. Twenty training images were preprocessed using a data augmentation approach, which is discussed in Section 2. Data for the first dataset was collected from June 6, 2020 to July 18, 2020 from a greenhouse for cherry tomato plants from the seedling stage to the flowering and fruit setting stages. The main pests were tobacco whiteflies, leaf miners, aphids, and fruit flies. Data for the second dataset was collected from March 1, 2021 to April 12, 2021 from a greenhouse for strawberry plants during the flowering and fruit-setting stages.

### 2.2 Data collection system

**Figure 2:**
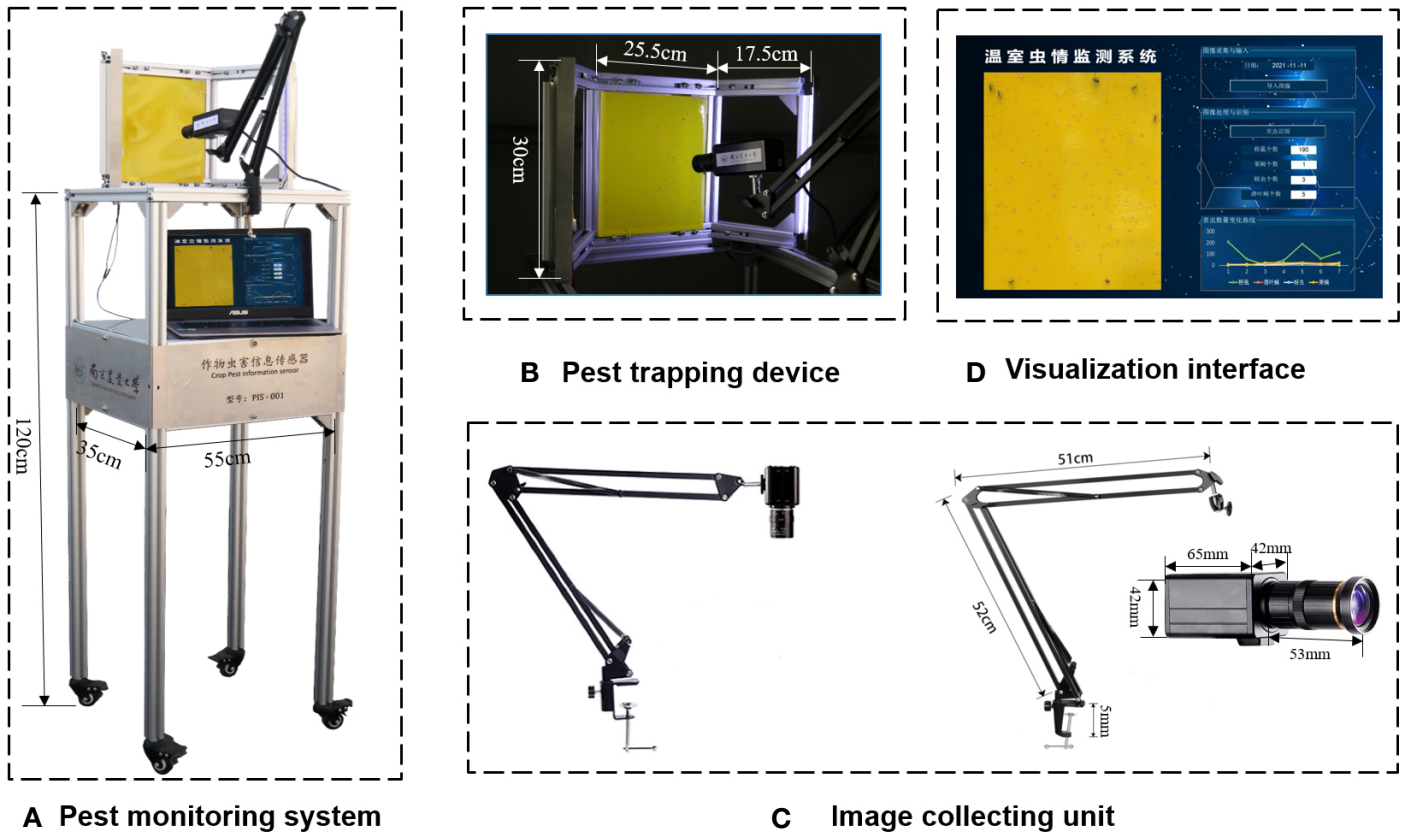
Automatic pest identification and monitoring system with LED trap lamp, sticky paper and image acquisition system. **(A)** Pest monitoring system, **(B)** Pest trapping device, **(C)** Image collecting unit, **(D)** Visualization interface.

An automatic pest identification and monitoring system with an LED insect trapping lamp and yellow sticky paper are established to attract pests and collect images in greenhouses. The system consists of a pest trapping device, a power supply unit, an image collecting unit, a pest data processing unit, and a visual interface (**Figure 2A**).

The pest-trapping device consisted of a stainless-steel plate, an aluminum profile frame, a yellow insect-trapping plate, and LED lamps (**Figure 2B**). The size of the yellow insect-trapping board was 20 × 25 cm, with a wavelength of 575 nm ± 10 nm. The wavelength of the LED insect-trapping lamp was 365 nm, and its voltage was 12 V. Two LED light strings were fixed on both sides of the frame, with one string consisting of 10 lamp beads. A storage battery with an output voltage of 12 V was used to provide 24-h power supply for the device. By adjusting the incident angle of the trapping device, the LED lamp could simultaneously trap the insects and provide light, making the trapping board images clear and conducive to the process. The pest-image collecting device consisted of an industrial camera and a cantilever bracket (**Figure 2C**). A Sony IMX226 camera with 3280*2464 resolution was installed in a cantilever and aimed at a piece of sticky paper at an appropriate distance to capture clear images. The visualization interface shows pest images and their location, types, and numbers (**Figure 2D**). The visualization interface presented dynamic changes in pest numbers within a week. Computation and visualization were conducted using Python on a Windows PC (Intel^®^ Core™ i7-7500U) with a RAM of 8 Gb.

### 2.3 Benchmark YOLOv5 model

**Figure 3:**
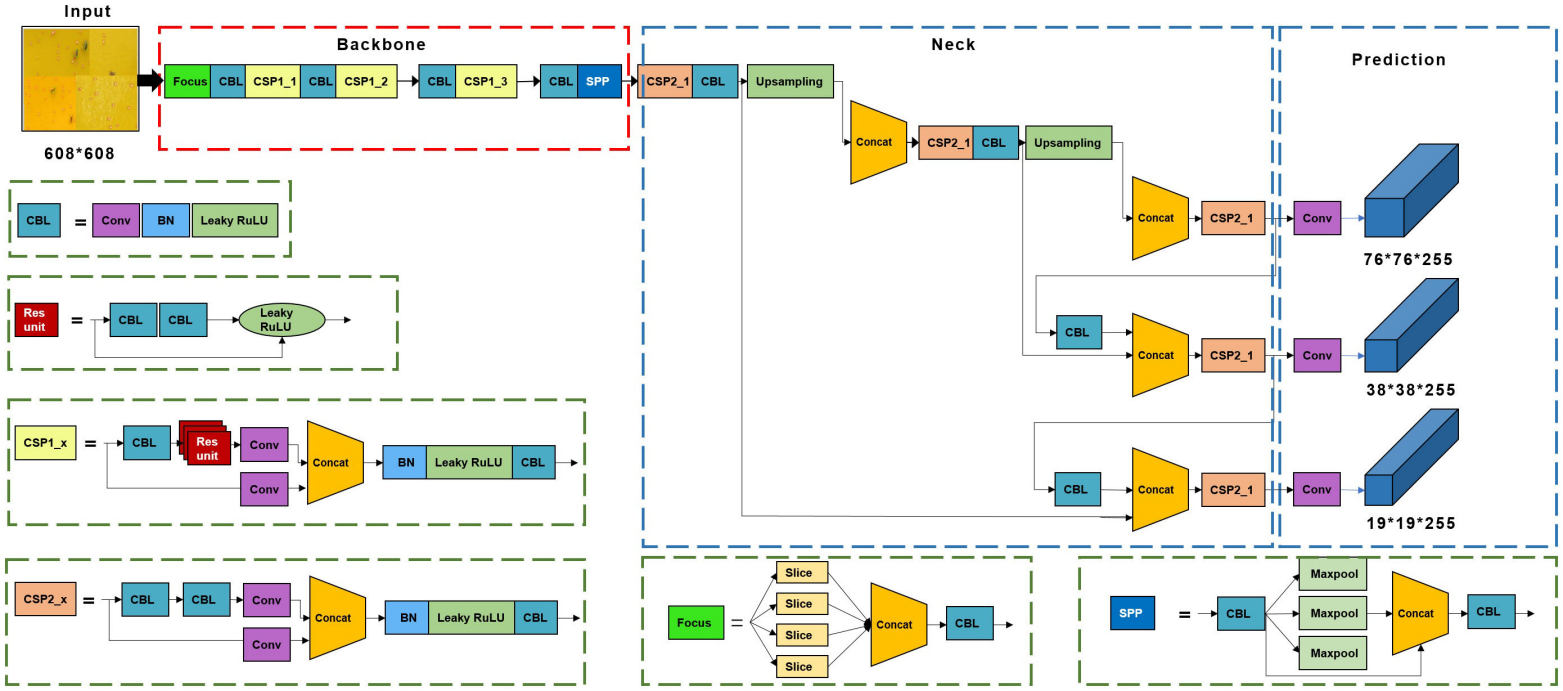
Structure of the YOLOv5 model for pest detection.

The benchmark model adopted in this study was YOLOv5 (**Figure 3**), which was released in 2020 for object detection. The YOLOv5 model consists of backbone, neck, and head modules that connect the procedure for predicting bounding boxes with class labels in an end-to-end differentiable network (Ultralytics, 2020). The YOLOv5 model has demonstrated excellent performance in small object detection in previous studies (Mathew and Mahesh, 2022; Zhan et al., 2022); thus, it was selected for identification of small-sized pests in this study.

#### 2.3.1 Mosaic data augmentation

**Figure 4:**
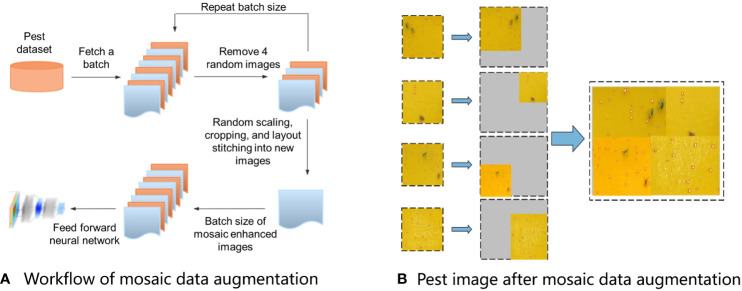
Mosaic data augmentation adopted in the YOLOv5 model. **(A)** Workflow of mosaic data augmentation, and **(B)** Pest image after mosaic data augmentation.

Mosaic data augmentation was adopted in the YOLOv5 model, which enriches the dataset by stitching together four images, thereby introducing novel object locations, partial occlusion, and variations in surrounding pixels for the model to learn from. The model could simultaneously process four images in the batch normalization layer, which decreased the GPU memory usage by using a relatively small mini-batch. The workflow of mosaic data augmentation was as follows: 1) random selection of four images from the original training dataset; 2) random rotation, scaling, flipping, and adjustment of the brightness and chromaticity of the four selected images; and 3) combining the images and box layout stitching into new images (**Figure 4A**) (Huang et al., 2022). The augmented images were enriched with various backgrounds, as shown in **Figure 4B**.

#### 2.3.2 Backbone

The backbone module extracted features from the input image and transmitted them to the neck module (**Figure 4**). The backbone includes the focus, CBL, Cross Stage Partial (CSP), and Spatial Pyramid Pooling (SPP) modules. The focus structure transformed the original 608×608×1 images into 304×304×32 feature maps using slicing and convolution operations, which increased the computational complexity, but retained the original features. The CBL comprises one convolutional layer, one batch normalization layer, and one Leaky ReLU layer. CSP1 was used in the backbone network, and CSP2 was used in the neck network. The SPP network outputs a feature map of fixed size with multiscale feature fusion through the 1×1, 1×5, 9×9, and 13×13 max pooling.

#### 2.3.3 Neck

The neck module generates a feature pyramid based on a path aggregation network (PANet) (Liu et al., 2018). This enables the model to detect pests of different sizes by shortening the information path between low-level spatial features and high-level semantic features through bottom-up path augmentation. Adaptive feature pooling directly propagates the effective information at each level to subsequent subnetworks (Liu et al., 2018).

#### 2.3.4 Head module

The head module provides detection boxes, pest categories, coordinates of the detected pests, and confidence values. The loss function in the head module includes classification loss and bounding box regression loss. The YOLOv5 model uses the complete intersection over union (CIoU) loss (Zheng et al., 2020), which improves the regression accuracy and convergence speed by considering the distance between the detection frame and target box, overlapping area, aspect ratio, and other aspects.

### 2.4 Improved YOLOv5 model

**Figure 5:**
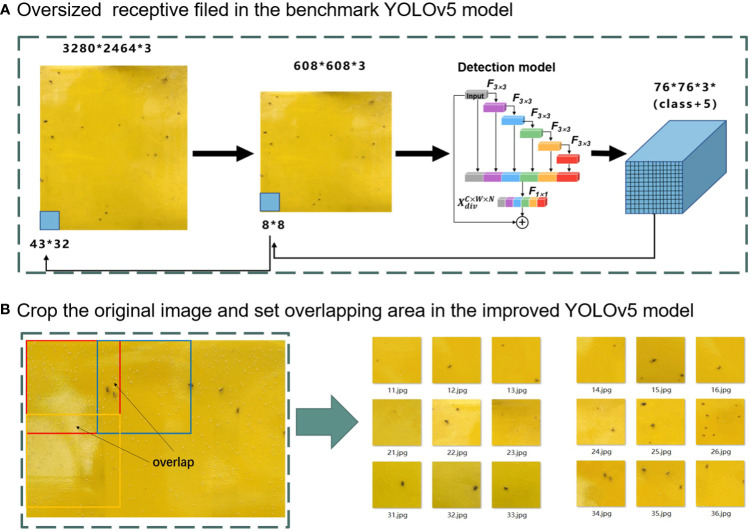
**(A, B)** Receptive field before and after copy-pasting data augmentation.

The mosaic data augmentation approach shows limitations on the proposed datasets where small targets comprise more than 80% of total pests. Mosaic data augmentation improves the accuracy of small target identification only if there are more large and medium targets than small targets. For example, in **Figure 5A**, the size of the raw image was 3280 × 2464 pixels. The images were resized to 608 × 608 pixels before being fed into the YOLOv5 network. Thus, the output feature sizes were 19 × 19, 38 × 38, and 76 × 76 pixels. The largest feature map (76 × 76) corresponded to the smallest anchor box for small target detection. Its receptive field was 8 × 8 when back-propagated to an input image of 608 × 608 pixels. When the 8 × 8 receptive field corresponded to the raw image, it was approximately 43 × 32 (**Figure 5A**). Therefore, insects smaller than 43 × 32 pixels in the raw image were not recognized in the YOLOv5 model. Therefore, the mosaic data augmentation approach reduces the recognition precision for tiny pests. To improve the pest identification performance of the proposed pest images collected from greenhouses, an improved YOLOv5 model adopting a copy-pasting data augmentation approach to virtually increase the number of pests is developed.

#### 2.4.1 Improved data augmentation approach

A copy-pasting augmentation approach was developed for data augmentation to improve the identification accuracy for small objects (**Figure 5B**). A raw image of 3280 × 2464 pixels was cropped by setting the overlapping area. The image was horizontally cropped into six pieces, each with 600 pixels, and the overlap length was set to 64 pixels. The image was also vertically cropped into 5 pieces, each piece of size 600 pixels, and the overlap length was set to 134 pixels. Therefore, the raw images were divided into thirty smaller images with 600 × 600 pixels. Setting the overlapping area could improve the detection accuracies for pests on the segmentation lines. Finally, to reduce the number of overlapping detection boxes, a non-maximum suppression (NMS) operation was performed on the entire image.

#### 2.4.2 Improved head module

**Figure 6:**
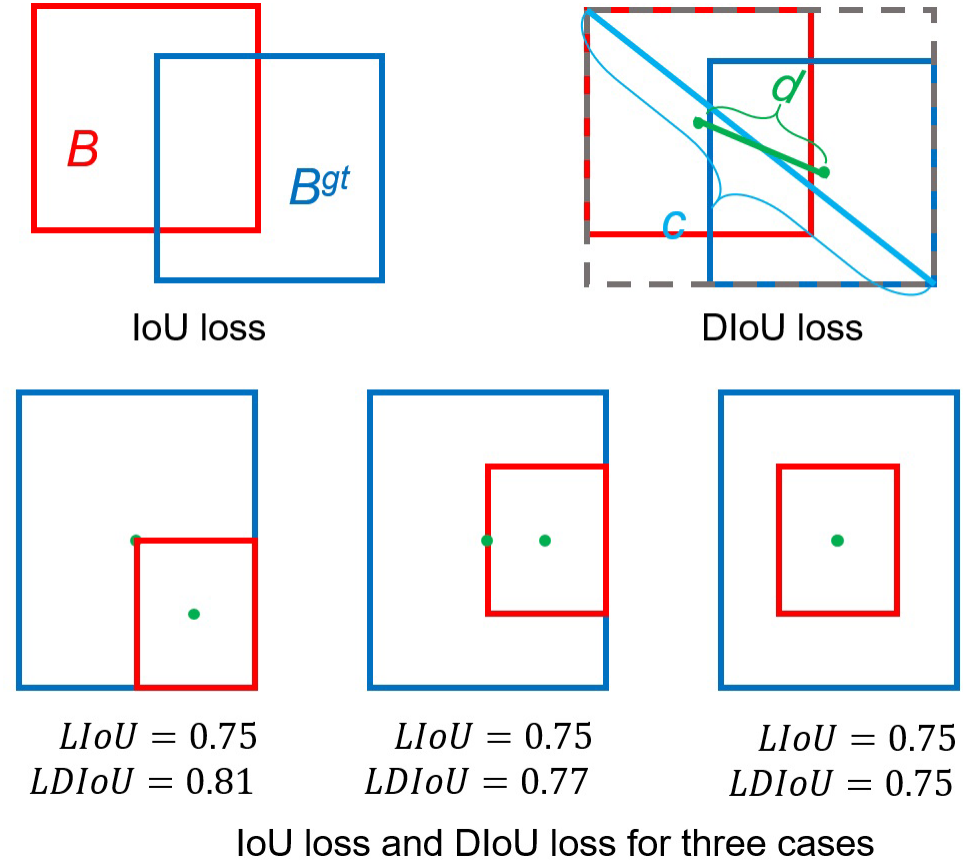
Intersection over union (IoU) and distance IoU losses and three application scenarios. (Blue and red colors represent the target box and predicted box, respectively.).


The YOLOv5 model generated candidate anchor boxes with various sizes and shapes, but these windows supposedly to contain one object, so it is necessary to filter out the ones. NMS is adopted to remove redundant boxes when their overlaps exceed a threshold. The intersection over union (IoU) loss is commonly used in NMS, but it demonstrates poor performances for the nonoverlapping boxes. The improved distance IoU (DIoU) considers the overlap area and distance between the central points of two bounding boxes when suppressing redundant boxes, making the model more robust to occlusion objects (Zheng et al., 2020). (**Figure 6**).

(1) $$LIoU\;=\;1\;-\;\frac{\left|B\cap^{}B^{gt}\right|}{\left|B\cup^{}B^{gt}\right|}$$

(2) $$LDIoU\;=\;1\;-\;IoU\;+R_{DIOU}$$

(3) $$R_{DIOU}=\;\frac{\rho^{2}\left(b,b^{gt}\right)}{c^{2}}$$

Where $B^{gt}$ is the ground truth, and B is the predicted box, The $R_{DIOU}$ is the distance between the center points of two   boxes, b and $\;b^{gt}$ represent the center points of the anchor frame and target frame respectively, the $\rho \left(b,b^{gt}\right)$ is the distance between the two center points, and c is the diagonal distance of the smallest rectangle covering two boxes.

In the improved YOLOv5 model, DIoU was deployed in the NMS and applied to the head module to remove redundant bounding boxes and improve the detection accuracy of occluded pests (**Figure 6**). The DIoU-NMS approach is more robust than the original NMS used in the YOLOv5 model for removing redundant boxes. Therefore, the DIoU-NMS method adopted in the improved YOLOv5 model improved the detection accuracy of occluded pests. The DIoU-NMS was defined as follows:


(4) $$S_{i}=\{\begin{array}{c} S_{i},IOU-R_{DIOU}\left(M,B_{i}\;\right)<\varepsilon \;\\ 0,IOU-R_{DIOU}\left(M,B_{i}\;\right)\geq \varepsilon \end{array}$$

where S is the confidence level of category i , ε is the threshold of the NMS, M is the box with the highest confidence level, and IOU is the intersection ratio of the anchor and target frames (Zheng et al., 2020).

All training and processes were implemented on a Python library torch 1.9.0 framework in the Pycharm platform with Python 3.6. The computations were performed on a Windows workstation with an Nvidia GeForce 940MX graphics card (NVIDIA Corporation, Santa Clara, California, United States).

### 2.5 Performance metrics

The model performance is evaluated by precision, recall, F1-score, and accuracy, which are shown in Eqs. (5) to (8).


(5) $$precision\;=\;\frac{tp}{tp+fp}$$

(6) $$recall\;=\;\frac{tp}{tp+fn}$$

(7) $$F1=\;2\times \frac{precision\times recall}{precision\;+recall}$$

Where the tp (true positive) represents the number of correct positive predictions, fp (false positive) represents the number of incorrect positive predictions, and fn (false negative) represents the number of incorrect negative predictions.

The mean average precision (mAP) is adopted for evaluating the object detection performance. It is calculated by taking the average of the AP scores across all classes. The AP value is obtained by calculating the area under the precision-recall curve, which measures the trade-off between precision and recall at different confidence thresholds.

### 2.6 Pest dataset preparation

**Figure 7:**
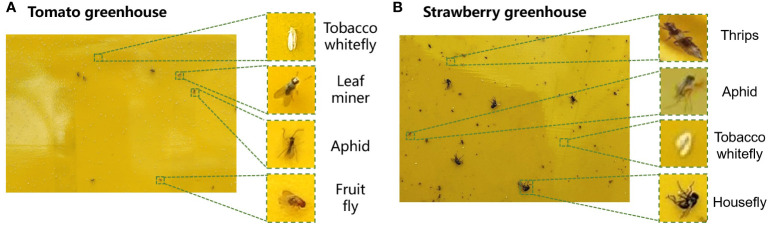
Images of pests collected in the **(A)** cherry tomato greenhouse and **(B)** strawberry greenhouse.

The datasets collected from the two greenhouses included six types of pests: tobacco whiteflies, leaf miners, aphids, fruit flies, thrips, and houseflies (**Figure 7**). Houseflies had the largest length of 5–8 mm while thrips were the smallest, with a length of 0.5–2 mm. Tobacco whiteflies were the highest in number but were difficult to detect by the human eye because of their white color. The lengths of the leaf miners, aphids, and fruit flies were 4–6 mm, 2.2 mm, and 1.5–4 mm, respectively. The number distribution of different types of pests are unbalanced, which brings challenge for the pest detection.

We collected 20 images with size of 3280 × 2464 pixels in the two greenhouses to train the YOLOv5 model. However, after data preprocessing with mosaic data augmentation, 450 images of size 600 × 600 pixels were acquired to train the insect pest detection model. Of the 450 training images, 200 were randomly selected for copy-pasting data augmentation. In total, 1,024 tobacco whiteflies, 857 thrips, 1,092 winged aphids, 941 leaf miners, 873 fruit flies, and 1,013 houseflies were included in the training set (**Table 2**).

**Table 2:** Pest dataset description.

	# images	# tobacco whiteflies	# thrips	# winged aphids	# leaf miners	# fruit flies	# houseflies	# total pests
Training set	450	1,024	857	1,092	941	873	1,013	5,800
Test set	80	5,832	2,928	1,001	460	890	267	12,014

The test set contained 328 images of 12,014 pests, including 5,832 tobacco whiteflies, 2,928 thrips, 1,001 winged aphids, 460 leaf miners, 890 fruit flies, and 267 houseflies (**Table 2**). The test set was used to monitor the pest population dynamics over a long period; therefore, the number of pests in the test set was larger than that in the training set.

## 3 Results and discussion

### 3.1 Pest detection results

**Figure 8:**
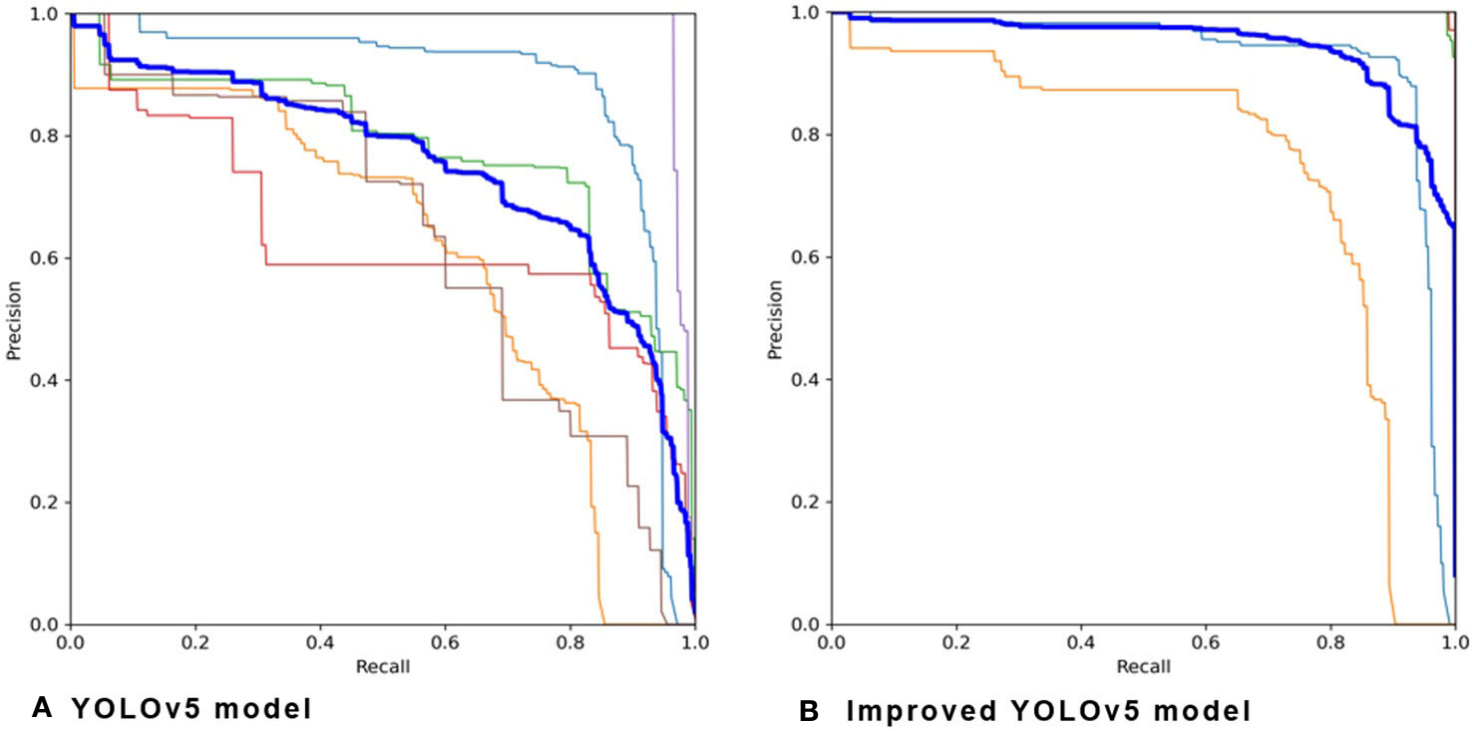
Precision-recall graph obtained on the **(A)** YOLOv5 model and **(B)** improved YOLOv5 model.

The improved YOLOv5 model obtained higher accuracy than the original YOLOv5 model by adopting copy-pasting data augmentation (**Table 3**). The precision-recall graph, depicted in **Figure 8**, compares the performance of YOLOv5 and the improved YOLOv5 models. The overall pest-detection precision improved from 64% to 96% by using the improved YOLOv5 model. The improved YOLOv5 model obtained the highest detection precision of 99% on leaf miners and fruit flies, followed by aphids and houseflies, with a precision of 98%. Thrips are very difficult to identify because of their small size. The improved YOLOv5 model obtains a precision of 83% for thrips, which is still higher than that of the YOLOv5 model (80%). The improved model achieved an F1 score of 0.99 for detecting aphids, leaf miners, fruit flies, and houseflies, and a score of 0.98 for detecting whiteflies. However, it exhibited a lower F1 score of 0.91 for detecting thrips. The primary reason for the low detection accuracy of thrips may be the varying size of trips during different growth stage. Image resolution may be another factor that some pests are confused with dust. Furthermore, the improved YOLOv5 model required more time for image processing because it adopts a copy-pasting operation and feeds the input images into the network in different batches, the recognition speed of the improved YOLOv5 model was lower than that of the original YOLOv5 model.

**Table 3:** The pest detection accuracies and average detection time of the models.

	YOLOv5 model	Improved YOLOv5 model
Average detection time (s/image)	0.83	7.53
Overall pest detection precision (%)	65	96
Tobacco whitefly detection precision (%)	92	97
Thrips detection precision (%)	80	83
Aphid detection precision (%)	56	98
Leaf miner detection precision (%)	41	99
Fruit fly detection precision (%)	88	99
Housefly detection precision (%)	30	98

**Figure 9:**
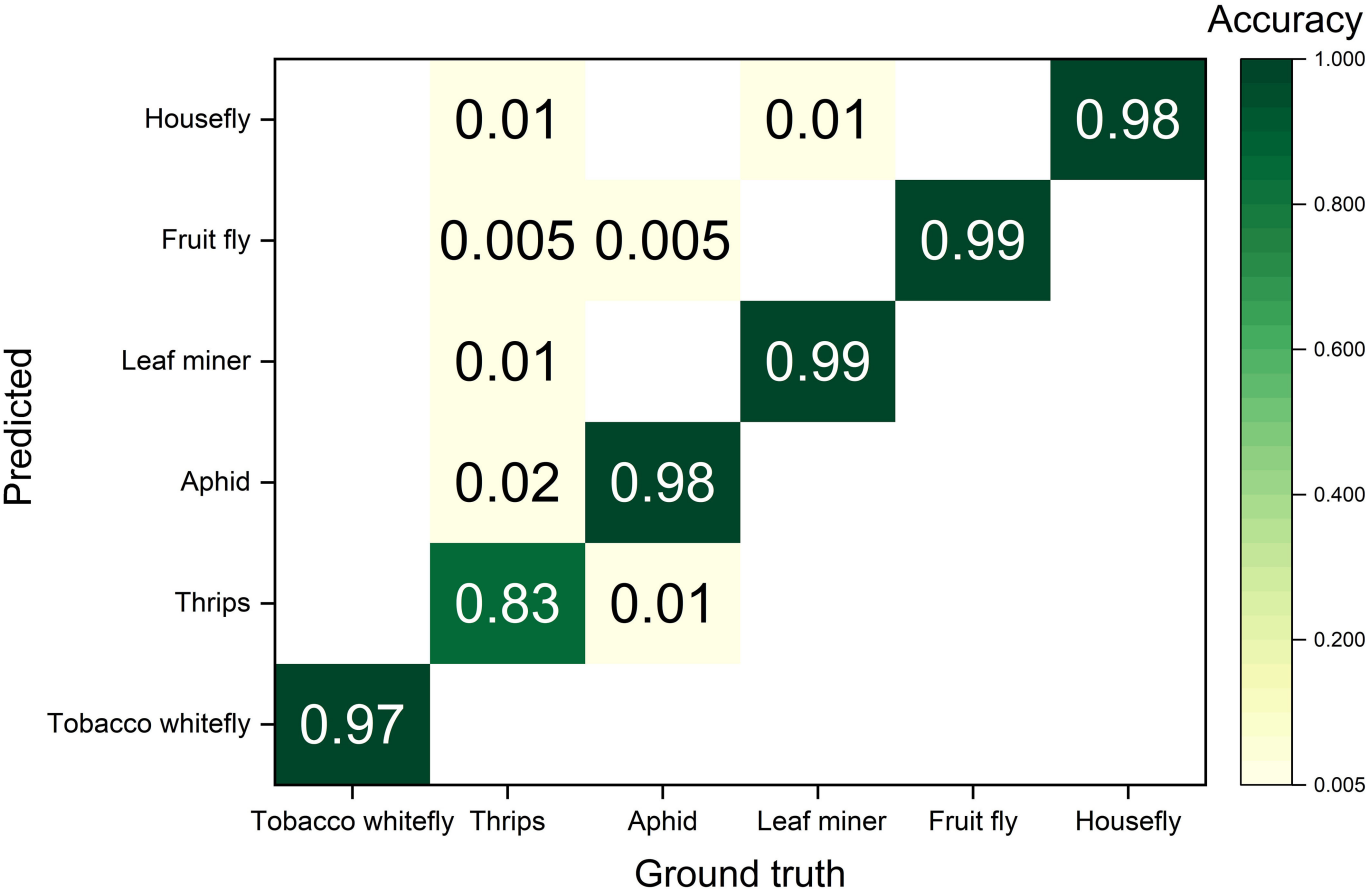
Confusion matrix of ground truth and predicted pests using the improved YOLOv5 model.

A confusion matrix of the detection accuracies is shown in **Figure 9**. The improved YOLOv5 model demonstrated the best performance for leaf miners and fruit flies, with a 99% detection accuracy. 1% of leaf miners were incorrectly recognized as thrips and 1% of fruit flies were incorrectly detected as fruit flies and aphids. The model showed 98% accuracy for aphids and houseflies, while 2% of the aphids were incorrectly recognized as thrips, 1% of houseflies were incorrectly recognized as thrips, and 1% of houseflies were incorrectly identified as leaf miners. Among tobacco whiteflies, 97% were correctly detected, and 3% were missed. The model obtained the lowest detection accuracy for thrips at 83% accuracy, with 1% being incorrectly recognized as aphids. Missed-detected pests were recognized as background.

**Figure 10:**
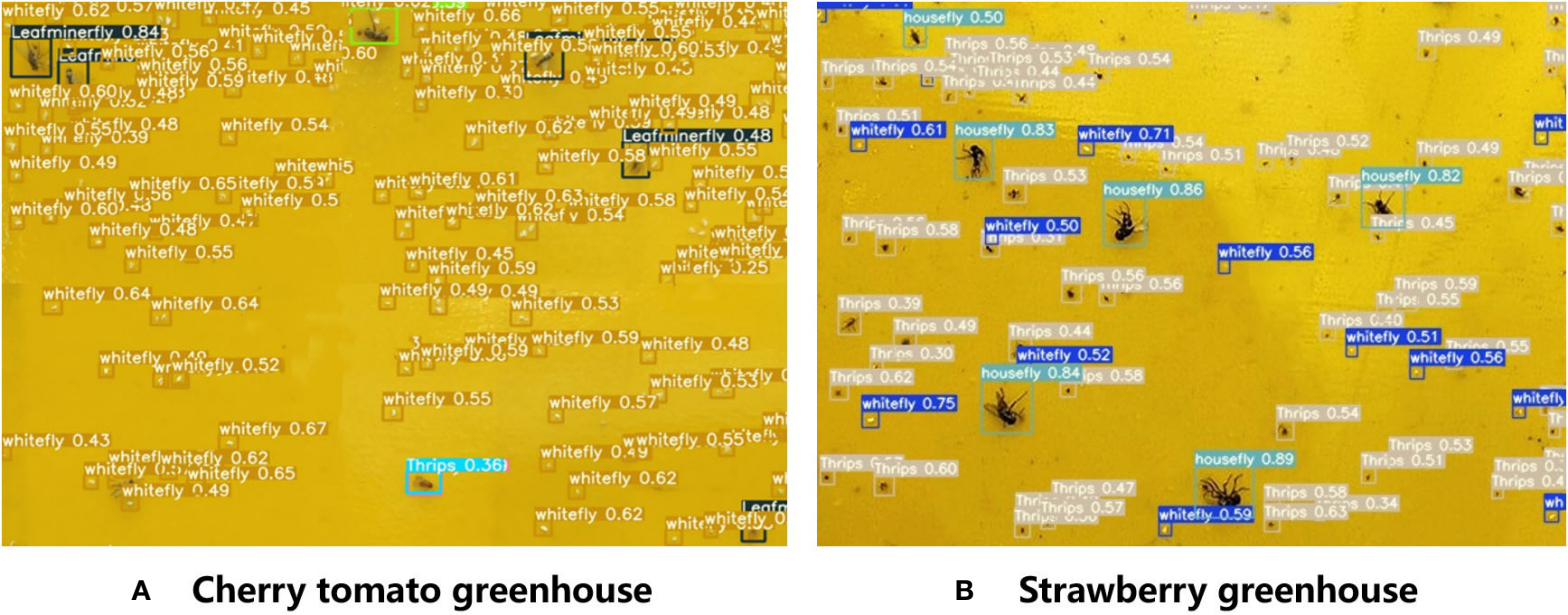
Results of pest detection for the two greenhouses using the improved YOLOv5 model. **(A)** Cherry tomato greenhouse, **(B)** Strawberry greenhouse.

**Figure 11:**
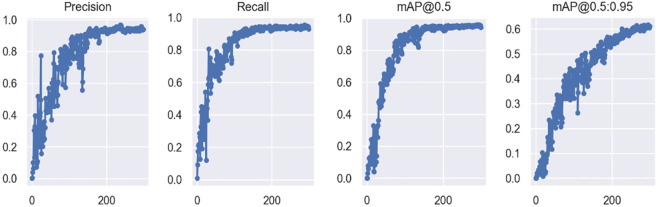
Plot of precision, recall and mAP after training the improved YOLOv5 model.

The recognition performance of the improved YOLOv5 model is illustrated in **Figure 10**. The improved YOLOv5 model demonstrated high classification accuracies for both cherry tomato and strawberry greenhouses. However, the model could not recognize tobacco whiteflies that were too light in color, and some tiny thrips were incorrectly recognized as small dust particles. Houseflies and leaf miners are similar in color and shape; therefore, they are sometimes misidentified. Precision, recall and mAP after training the improved YOLOv5 model are shown in **Figure 11**.

### 3.2 Recognition performance on adjacent pests

**Figure 12:**
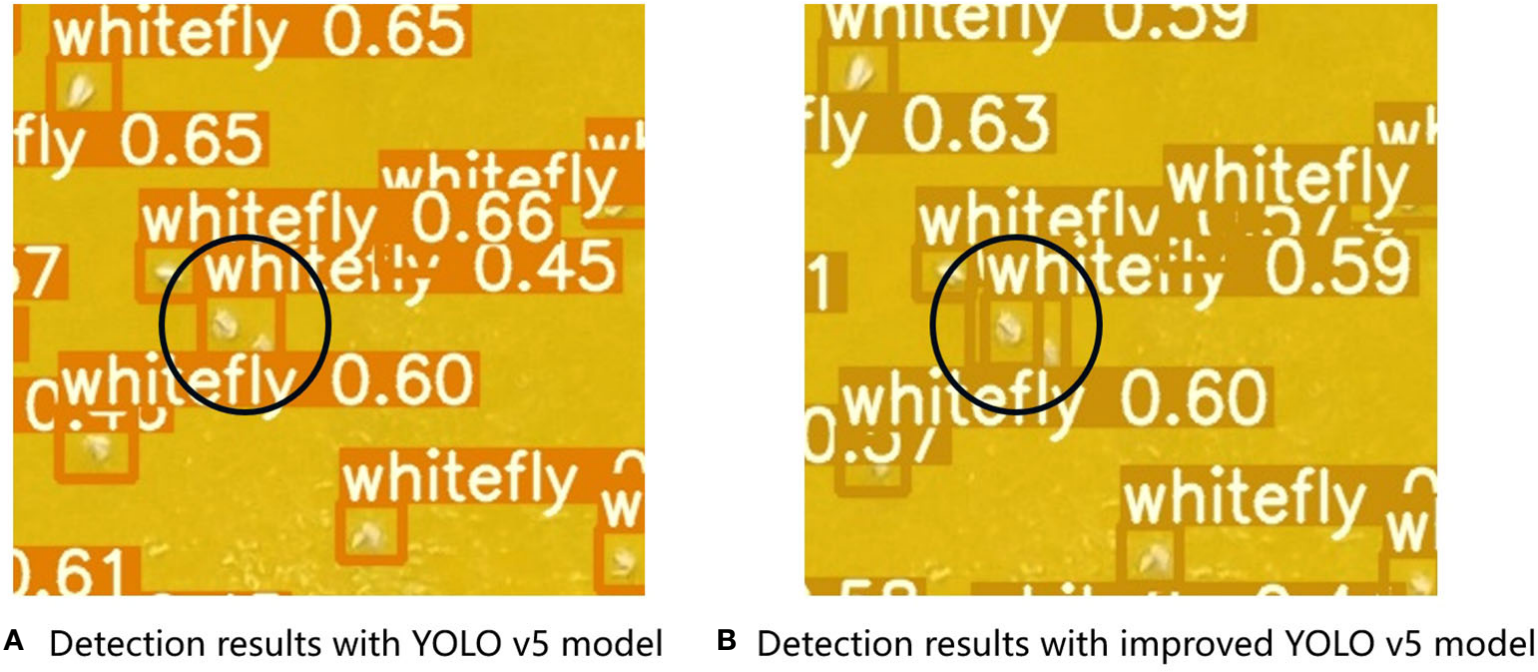
Pest recognition performance on two adjacent tobacco whiteflies using **(A)** YOLOv5 and **(B)** improved YOLOv5 models.

The improved YOLOv5 model demonstrated better identification accuracy for nearby insects and could distinguish between two adjacent whitefly insects (**Figure 12**). For example, the original YOLOv5 model recognized two tobacco whiteflies as one pest in the black circle, whereas the improved YOLOv5 model could distinguish two adjacent whiteflies. DIoU-NMS computes the overlapping area of pests and the central point distance between two pest boxes when suppressing redundant boxes.

### 3.3 Real-time pest population dynamics in the cherry tomato and strawberry greenhouses

The improved YOLOv5 model obtained reliable prediction results; the predicted number of pests showed similar trends to the manual counting results. The cherry tomato greenhouse had a total of 7619 pests after 40 days of continuous monitoring as compared to 4395 pests in the strawberry greenhouse.

**Figure 13:**
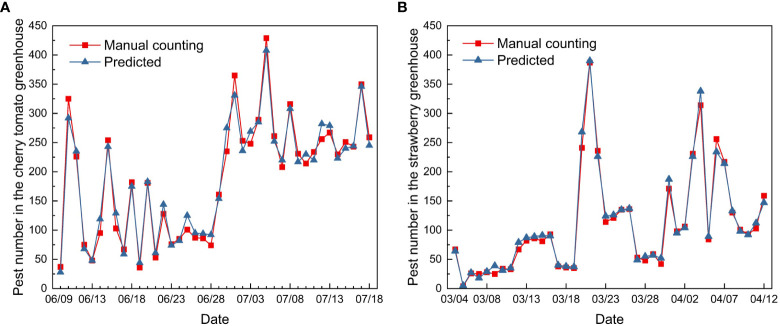
Pest population dynamics in the **(A)** cherry tomato greenhouse and **(B)** strawberry greenhouse during the 40 days of monitoring.

The dynamic trends in the two greenhouses demonstrated different patterns at different growth stages of cherry tomatoes and strawberries (**Figure 13**). The number of pests in the cherry tomato greenhouse increased sharply from 37 to 325 from June 9th to 10th. Thereafter, the number of total pests decreased to 92 on June 28th, and rose rapidly in late June and early July. The number of pests was at its highest from 214 to 419, from July 1st to July 18^th^, although there were fluctuations. The strawberry greenhouse had a low density of pests from March 4th to March 19th, and showed two peaks on March 23rd and April 3rd. Changes in pest numbers may be related to environmental temperature, humidity, and the growth stages of greenhouse crops (Aiello et al., 2018). Understanding pest outbreaks may help identify periods of risk in greenhouses and provide decision-making support for managers.

**Figure 14:**
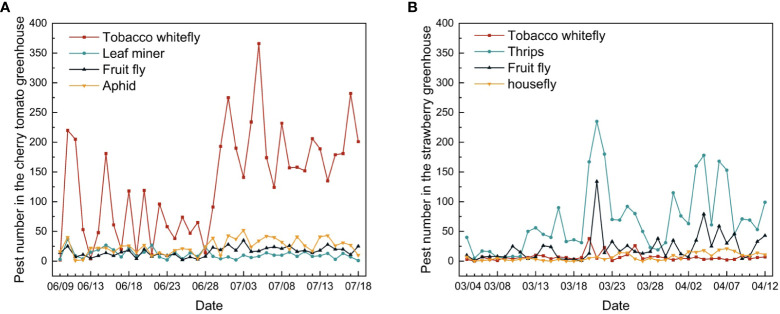
Changes in individual pest numbers in the **(A)** cherry tomato greenhouse and **(B)** strawberry greenhouse during the 40 days of monitoring.

Pest diversity and frequency varied between the two greenhouses (**Figure 14**). The main pest observed in the cherry tomato greenhouse was the tobacco whitefly, whereas thrips were the most prevalent pest in the strawberry greenhouse. The number of tobacco whiteflies in the cherry tomato greenhouse was approximately 2.5 times the total number of leaf miners, fruit flies, and aphids. In the strawberry greenhouse, 87% of the pests comprised thrips, fruit flies, tobacco whiteflies, and houseflies. In the cherry tomato greenhouse, the dynamic changes in tobacco whitefly, fruit fly, and aphid showed patterns similar to the overall trend of pest numbers, which decreased in June and increased in July. The population trend of leaf miners showed the opposite pattern, increasing in June and decreasing in July. In the strawberry greenhouse, the numbers of thrips and fruit flies showed the same trend of increasing in late March and early April. The numbers of tobacco whiteflies and houseflies fluctuated and showed a small increase at the same time. The dynamic trend in pest numbers provides insights into pest control during certain periods throughout the year, which is of vital importance for greenhouse management.

Obtaining the dynamic changes in pest occurrence will help us to further build pest prediction models based on time series and understand the patterns of greenhouse pest occurrence, thus assisting agricultural pest control decisions (Chen et al., 2022). The present study monitored pest populations in two greenhouses for 40 days, which provided the change in number of pests at different times. This study provides insights for pest management at different plant growth stages.

## 4 Conclusions

This study proposed a pest detection and long-term pest number monitoring system for cherry tomato and strawberry greenhouses. To obtain high-quality images, we designed a sticky board trap that combines yellow sticky paper and LED pest-trap lamps to achieve all-weather pest-trapping effects. The LED lamp can also be used as a light supplement to increase the brightness of the trap board image and alleviate the problem of uneven illumination, thereby improving image quality. The pest image capturing system with LED traps provided clearer images compared to that adopted sticky paper only. The system was applied to cherry tomato and strawberry greenhouses, and the improved YOLOv5 model obtained an overall pest detection precision of 96% during the 40 days of monitoring. The model achieved the highest F1 score of 0.99 for the detection of four types of pests, while the lowest F1 score of 0.91 was obtained for thrips. This system provides important decision-support information for the management of pests and diseases in greenhouses. The pest-monitoring system developed in this study can be applied to other types of greenhouses for pest image collection and for building pest-detection models for a large number of common pests. The system can also be used for pest population dynamics and status prediction, considering future changes in climate and weather conditions.

## Data availability statement

The raw data supporting the conclusions of this article will be made available by the authors, without undue reservation.

## Author contributions

XLZ: Conceptualization, Writing – review & editing, Methodology, Writing – original draft. JB: Conceptualization, Data curation, Methodology, Software, Writing – original draft, Writing – review & editing. XXZ: Writing – original draft, Writing – review & editing. XW: Funding acquisition, Investigation, Supervision, Conceptualization, Writing – review & editing, Data curation.
